# Psychosocial care and support in the field of intersex/diverse sex development (dsd): counselling experiences, localisation and needed improvements

**DOI:** 10.1038/s41443-021-00422-x

**Published:** 2021-03-16

**Authors:** Ute Lampalzer, Peer Briken, Katinka Schweizer

**Affiliations:** 1 Institute for Sex Research, Sexual Medicine and Forensic Psychiatry, University Medical Center Hamburg-Eppendorf, Hamburg, Germany; 2 Department of Psychology, Medical School Hamburg, Hamburg, Germany

**Keywords:** Therapeutics, Quality of life

## Abstract

From different sides, there is a call for better psychosocial care and counselling in the field of diverse sex development (dsd). However, studies on the specific demands, deficits and needed improvements regarding those services are rare. This exploratory online study aimed at investigating counselling experiences and the ideas that different groups of participants have concerning the localisation of counselling structures and improving care. Quantitative and qualitative data (*N* = 630) were analysed within a mixed methods framework. The participants included experts of experience resp. patients with different intersex/dsd conditions (*n* = 40), parents of children with dsd (*n* = 27), professional psychosocial counsellors (*n* = 321) and experts in the field including medical practitioners, psychologists, natural and social scientists as well as others involved, e.g., students or relatives (*n* = 56). The results show a gap between receiving psychosocial and medical care in the group of adult lived-experience experts, who had received less psychosocial care than medical interventions. The findings also reveal important tasks of psychosocial care. A focus was set on parental experiences. Helpful aspects reported were talking with other parents of children with intersex/dsd, aspects missed were assistance in supporting the individual development of their children. The majority of all participants (58%) held the view that, apart from multidisciplinary competence centres, there also have to be easily accessible counselling services which offer support in everyday life. The participants named increasing quality and quantity as necessary improvements in counselling structures for children and adults with intersex/dsd and their families. Implications are drawn for the specific tasks and target groups of psychosocial care and needed research in intersex healthcare over life span.

## Introduction

From different sides, there has been a call for better psychosocial care and counselling for individuals with different forms of diverse sex development (dsd) and parents of children with intersex/dsd conditions [1–8]. But empirical data on existing counselling services, on specific counselling demands, deficits and resources are scarce. Research has specifically shown a great need of psychosocial support to encourage self-acceptance, process trauma, accept the experienced otherness and improve sexual satisfaction, as well as overall psychosocial well-being. Often, intersex/dsd conditions and diagnoses are accompanied by adaptation and self-esteem problems, infertility and/or insecurities regarding gender identity, heightened sexual fear, problems of sexual communication and dissatisfaction with sexual functioning and outer appearance of the genitalia [9–17]. Children and families affected were described as being exposed to shame and fear of negative reactions, which can lead to social withdrawal and isolation [11, 15, 17, 18].

In the past, medical interventions within the context of “Optimal Gender Policy” were often accompanied by stressful situations such as long hospital stays, pain, missing freedom of choice, longstanding concealment and insensitive communication of the diagnosis later on [10, 17–19]. Given the paradigmatic change towards a full informed consent approach, treatment policies and ethics are changing. Research and human rights activism both contributed to an altered perspective that stresses the importance of long lasting psychosocial support; psychosocial care and peer counselling are now central elements requested by the new guidelines [20–22].

From the viewpoint of the experts in the field, appropriate care for persons with intersex/dsd and their parents should include interdisciplinary teams with effective communication structures and joint efforts regarding goals, settings, values and visions [23–25]. These structures should guarantee open and empathetic professional interactions and communication [5, 7]. Parents themselves have reported to seek help with finding words for the conditions they are newly faced with and with dealing with intersex in the family [26, 27]. Information management on conditions and medical implications has been described as being too stressful to appropriately process the information in the first place, and therefore being processed and coped with rather at a later point in time. Furthermore, parents address the need for a continuous psychological support that offers a safe space to enable coping and adjustment, and is addressing fear (e.g., considering the child’s future and stigmatisation) and psychological stress as well as preventing premature decisions [28–32]. How to reach full informed consent and how to address minor consent have been reported to be controversial issues in these processes [27]. In addition, the need of encounter and exchange with other parents in a similar situation and with similar experiences has been addressed [28]. So far, there are almost no studies that directly asked different target groups about the deficits and needed improvements regarding counselling services and psychosocial care within the field of intersex/dsd.

### Research questions

This paper addresses needs, deficits and improvements for psychosocial care and support structures in the field of intersex/dsd with a focus on parental experiences. It presents data from a larger study on psychosocial care structures in the field of intersex/dsd in Germany. Here we address the following research questions:

- Counselling experiences and preferences: Which experiences, needs (e.g., contents and situations) and preferences of counselling do adult experts of experience and parents of children with intersex/dsd report? A focus is set on parental preferences.
- Localisation of counselling offers: Where should help structures, particularly the establishment of multidisciplinary competence and expert centres for children and adolescents, be located?
- Improving care: In which areas do the participants see the main needs for improvement?

## Method

### Methodology and measures

For this explorative online study, a mixed methods design was chosen to examine the research questions. The study is part of a project that was conducted as an online survey on behalf of the Federal Ministry for Family Affairs, Senior Citizens, Women and Youth of Germany (BMFSFJ). The study was approved by the local ethics committee in Hamburg.

A questionnaire was developed that is suited to be answered by different groups of participants. It covers different aspects and issues relevant to healthcare research, e.g., demand, utilisation, organisational, structural and quality aspects. Most of the questions are single or multiple choice questions with predefined answer options. In addition to the quantitative part, the questionnaire also contains some open questions that could be analysed via qualitative content analyses. The present study used the software EFS Survey 10.5 by QuestBack AG.

The study addressed the following target groups: (1) adults with different intersex/dsd conditions, (2) parents of children with intersex/dsd, (3) psychosocial counsellors, including employees and heads of counselling centres (also those with less or no experience regarding intersex/dsd were explicitly encouraged to participate), (4) other professionals, such as medical practitioners, psychologists, social workers, lawyers and researchers. The participants were informed that taking part in the study was voluntary, that the data protection regulations were observed and that anonymity of the collected data was ensured. Completion of the online survey took ~35–40 min. The data collection took place between 31 July and 1 September 2015. There was no reward for participating in the study. While general findings have been published in German [33], more specific findings will be presented first time here.

### Data analysis

Quantitative data were analysed descriptively. Statistical analyses were done by means of the Statistical Package for the Social Sciences Version 22.0.

Qualitative data were analysed via qualitative content analysis referring to Mayring [34]. Categories were built inductively, i.e., via generalisations directly from the text without being based on previously defined theoretical concepts. The categories were built by the first author and, for cross-validation, in a triangulation process discussed with the last author and adjusted, e.g. refined or integrated into another category, if required. Due to publication purposes, open answers, which had been previously stated in German, were translated into English by the third author.

## Results

### Participants

Participants were recruited via e-mail invitations to participate in the study that were sent to nationwide advice centres (e.g., pro familia, child guidance offices, crisis counselling centres), expert groups, self-help and interest groups (e.g., “Intersexuelle Menschen e.V.”, CAH/“AGS-Initiative”, OII) as well as different professional networks, using different listservs and institutional addresses. Furthermore, a call for participation was published on the website of the authors’ institute at the University Medical Centre Hamburg-Eppendorf (UKE). During the data collection period, the weblink was called 1787 times. In total, 727 persons, i.e. 41% of the persons who had called the link, responded to the first question. Data of *N* = 630 persons were included in the analysis.

Table 1a depicts the backgrounds of the respondents, including the professional backgrounds of the professional experts, as well as the intersex/dsd conditions of the experts by experience. The participants were aged 18 years or above, they belonged to one of the four target groups and responded to the first general part of the survey. Of those, 6% (40/630) were lived-experience experts, 4% (27/630) were parents, 41% (256/630) worked at a counselling centre, 18% (115/630) were heads of a counselling centre, 22% (136/630) were experts (in the field) and 9% (56/630) belonged to the category “other”.

**Table 1:** a: participants and subgroups; b: adult experts with personal experience and parents: experiences with specific intersex/dsd conditions, medical interventions and psychosocial care^e^; c: professional counsellors’ and experts’ counselling encounters and experiences with persons with intersex/dsd and/or their parents.

(a)			
Subgroups	*N* (%)	*N* (%)	Age MD (range) years
(1) Adult patients and experts with personal experience, i.e., intersex/dsd		40 (6.3)	45 (18–68)
Congenital adrenal hyperplasia (CAH)	3 (7.5)		
Androgen biosynthesis deficit	2 (5.0)		
Complete androgen insensitivity syndrome (CAIS)	12 (30.0)		
Partial androgen insensitivity syndrome (PAIS)	3 (7.5)		
Gonadal dysgenesis	5 (12.5)		
Klinefelter syndrome	5 (12.5)		
Turner syndrome	2 (5.0)		
Ovotesticular dsd	1 (2.5)		
Other (46, XY)	1 (2.5)		
Unknown (I do not know)	2 (5.0)		
No answer	4 (10.0)		
(2) Parents of a child with intersex/dsd		27 (4.3)	44.5 (32–85)
CAH	0 (0.0)		
Androgen biosynthesis deficit	0 (0.0)		
CAIS	4 (14.8)		
PAIS	1 (3.7)		
Gonadal dysgenesis	2 (7.4)		
Klinefelter syndrome	2 (7.4)		
Turner syndrome	4 (14.8)		
Ovotesticular dsd	2 (7.4)		
Unknown intersex/dsd	3 (11.1)		
Other (e.g., chromosomal mosaic, clitoris hypertrophy, gene defect)	4 (14.8)		
Unknown (we do not know)	1 (3.7)		
No answer	4 (14.8)		
(3) Professional counsellors		371 (58.9)	50 (24–64)
Social workers	149 (40.2)		
Psychologists	63 (17.0)		
Educators	40 (10.8)		
Medical doctors/practitioners	5 (1.3)		
Midwives	1 (0.3)		
Other professions (e.g., nurse)	42 (11.3)		
No response	71 (19.1)		
(4) Professional experts in the field of intersex/dsd^a^		136 (21.6)	47 (26–72)
Medical doctors/practitioners^b^	38 (27.9)		
Psychologists	32 (23.5)		
Social workers	10 (7.4)		
Educators	4 (2.9)		
Lawyers	2 (1.5)		
Preschool teachers	1 (0.7)		
Midwives	1 (0.7)		
Other professions (e.g., sociologists, historians, natural scientists)	16 (11.8)		
No response	32 (23.5)		
(5) Others (e.g., family members, students)^c^		56 (8.9)	46 (21–66)
Total all participants		630 (100)	48 (18–85)

(b)														
	Diagnoses					Medical interventions			Psychosocial counselling and care					
	Classification								Until now			Currently		
	46, XX	46, XY	Sex chr	Unkn^d^	n.a.	Yes	No	n.a.	Yes	No^f^	n.a.	Yes	No	n.a.
	*N* (%)	*N* (%)	*N* (%)	*N* (%)	*N* (%)	*N* (%)	*N* (%)	*N* (%)	*N* (%)	*N* (%)	*N* (%)	*N* (%)	*N* (%)	*N* (%)
Adult experts of experience														
(*N* = 40)	3 (7.5)	23 (57.5)	4 (10.0)	4 (10.0)	6 (15.0)	33 (82.5)	3 (7.5)	4 (10.0)	21 (52.5)	16 (40.0)	3 (7.5)	13 (32.5)	6 (15.0)	2 (5.0)
Parents of a child with intersex/dsd														
(*N* = 27)	1 (3.7)	13 (48.1)	3 (11.1)	5 (18.5)	5 (18.5)	15 (55.6)	9 (33.3)	3 (11.1)	15 (55.6)	11 (40.7)	1 (3.7)	12 (44.4)	3 (11.1)	0 (0.0)

(c)		
	Professional counsellors (*n* = 371)	Professional experts in the field (*n* = 136)
	*N* (%)	*N* (%)
Counselling experience with individuals with intersex/dsd or their parents		
Yes	76 (20.5)	63 (46.3)
No	280 (75.5)	63 (46.3)
No answer	15 (4.0)	10 (7.4)
Specific intersex/dsd counselling services		
Yes	14 (3.8)	32 (23.5)
No	305 (82.2)	86 (63.2)
No answer	52 (14.0)	18 (13.2)
Target groups of their service/offer	(*N* = 14)	(*N* = 32)
Parents of children with intersex/dsd	11 (78.6)	23 (71.9)
Children and/or adolescents with intersex/dsd	9 (64.3)	16 (50.0)
Adults with intersex/dsd	9 (64.3)	29 (90.6)
Family members, relatives	10 (71.4)	16 (50.0)

^a^Working in these institutions: clinic (4.4%, 6/136); university clinic (19.9%, 27/136), private practice/clinic (15.4%, 21/136), clinical centre (4.4% 6/136); other (44.9%, 61/136), no response (6.6%, 9/136).

^b^Including paediatricians, endocrinologists, urologists, gynaecologists, geneticists.

^c^These participants were asked about their involvement with the field of intersex/dsd. The replies included: institutional work, relatives of intersex/dsd people as well as one person with hypospadias and cryptorchidism (46, XY) and one person with CAH late onset, who did not perceive themselves as a person with dsd.

^d^These participant replied not to know the classification; the results differ from the conditions, addressing the issue of terminology as an important aspect in information management.

^e^Due to or in relation to their intersex/dsd condition, resp. their child’s intersex/dsd condition. Medical interventions included.

^f^Four adults replied “no, because there was no need for counselling”, 12 replied “no, because I did not know where to go/who was in charge”.

The expert group consisted of medical practitioners (28%, 38/136), psychologists (24%, 32/136), social workers (7%, 10/136), educators (3%, 4/136), lawyers (1%, 2/136) as well as preschool teachers (1%, 1/136) and midwives (1%, 1/136) and “others” (12%, 16/136). Twenty-four per cent (32/136) did not provide information on their professional background.

Eighty-two per cent (517/630) of all participants provided information on their age. The median age was 48 years (range: 18–85). In total, 57% (358/630) of the participants identified their current gender roles as female, 20% identified as male (125/630), 3% as “other” (19/630), 1% (9/630) as intersex and 19% (119/630) did not provide information. The experienced gender identity was distributed as the following: 48% (304/630) experienced themselves as female, 16% (103/630) experienced themselves as male, 16% (103/630) experienced themselves as non-binary, i.e. not exclusively female or male, and 19% (120/630) did not provide information on their experienced gender identity.

The most frequent educational background was high school graduation (56%, 354/630), followed by advanced technical college certificate (19%, 118/630, general certificate of secondary education (3%, 21/630) and certificate of secondary education (2%, 10/630). Two per cent (12/630) of the participants did not come from Germany, but from Austria or Switzerland, for example.

Table 1b presents experiences with medical interventions and psychosocial care of the adult experts of experience and the children of the parents who participated. While 83% of the adult experts of experience (33/40) had reported medical interventions due to their intersex/dsd conditions, only 53% (21/40) of them reported to have had counselling or psychosocial care. From the parents’ group (*n* = 40), 56% (15/27) reported their child had medical interventions, while the same number of 56% also reported to have received counselling as parents.

Table 1c gives an overview of the counselling experiences of the professional counsellors, e.g., in general psychosocial family counselling centres and the professional experts in the field. While 20% (76/371) of the counsellors reported to have had personal counselling experiences with individuals with intersex/dsd or their parents, 63% of the intersex/dsd experts had personal counselling experiences and encounters with patients or clients with intersex/dsd or their parents. In the group of professional counsellors only 4% (14/371) reported to provide a specific intersex/dsd counselling offer, while 24% (32/136) of the experts reported to offer such a specific counselling.

The participants got to know about the study in different ways. The majority directly received an e-mail invitation (47%, 297/630), 23% (145/630) were recruited via internet, 6% (39/630) via self-help groups, 5% (30/630) via intersex interest groups, 3% (17/630) via friends, 1% (4/630) via medical personnel and 16% (98/630) via other pathways.

### Counselling experiences

Table 2a presents the types of counselling in response to their or their child’s intersex/dsd condition that the personal experts resp. patients and parents had reported. The findings refer to those participants in both groups, who had reported personal counselling experiences, thus they only include a subgroup of parents (15/27) and adult experts of experience (21/40). In both groups, self-help groups were named most frequently by parents and the adult patients resp. experts of experience, followed by counselling from medical specialists, psychotherapy and psychological counselling. Both groups reported long distances to reach these counselling offers and structures, 57% of the adults and 53% of the parents reported to travel more than 200 km. The table also shows the different types of medical interventions.

**Table 2:** a: types of counselling, distance and types of medical interventions in relation to intersex/dsd; b: helpful and missed aspects in previous counselling experiences.

(a)		
	Adult experts of experience (*n* = 40) (*n* = 21 with counselling experiences)	Parents (*n* = 27) (*n* = 15 with counselling experiences)
	*N* (%)	*N* (%)
Counselling and support		
Self-help groups	19 (90.5)	14 (93.3)
Counselling from medical specialists (e.g., endocrinologist, gynaecologist, urologist)	12 (57.1)	13 (86.7)
Multidisciplinary team (MDT) in a reference centre/network	0 (0.0)	7 (46.7)
Psychotherapy	9 (42.9)	3 (20.0)
Psychological counselling	5 (23.8)	6 (40.0)
Peer-to-peer counselling	4 (19.0)	2 (13.3)
Counselling from my physical examiner (P.E.) (Hausarzt)	3 (14.3)	2 (13.3)
Legal advice	3 (13.6)	3 (20.0)
General counselling centre	0 (0.0)	1 (6.7)
Specialised counselling centre	2 (9.5)	0 (0.0)
Distance to obtain counselling (How far did you have to travel?)		
<25 km	3 (14.3)	3 (20.0)
<50 km	4 (19.0)	1 (6.7)
Max 100 km	2 (9.5)	3 (20.0)
More than 200 km	12 (57.1)	8 (53.3)
Medical interventions		
Hormonal replacement	26 (65.0)	7 (25.9)
Gonadectomy	24 (60.0)	6 (22.2)
External genital surgery	9 (22.5)	1 (3.7)
Internal genital surgery (e.g., vaginoplasty)	8 (20.0)	2 (7.4)
Other (e.g., hysterectomy, biopsy, hernia surgery, gonadal replacement)	7 (17.5)	6^a^ (22.2)
No answer	4 (10.0)	3 (11.1)

(b)		
	Adult experts of experience	Parents
	(*n* = 21)^b^	(*n* = 15)^b^
	*N* (%)	*N* (%)
Helpful aspects		
1. Talking with other persons with an intersex/dsd condition	17 (81.0)	9 (60.0)
2. Talking with other parents of children with intersex/dsd conditions	8 (38.1)	14 (93.3)
3. Understanding and emotional support	14 (66.7)	9 (60.0)
4. Broadening of my thinking	12 (57.1)	10 (66.7)
5. Community, solidarity and overcoming isolation	12 (57.1)	8 (53.3)
6. I have received important medical information.	8 (38.1)	8 (53.3)
7. I have received important information regarding sex and gender development.	8 (38.1)	7 (46.7)
8. Acknowledging my body resp. the body of my child	9 (42.9)	4 (26.7)
9. Supporting my individual development resp. of my child	8 (38.1)	6 (40.0)
10. I have received important legal information.	8 (38.1)	3 (20.0)
11. Support in decision-making	7 (33.3)	5 (33.3)
12. Minimising the pressure to decide (e.g., regarding irreversible medical interventions)	3 (14.3)	3 (20.0)
13. Other reasons^c^	3 (14.3)	1 (6.7)^c^
Aspects missed/wished for		
1. Talking with other persons with an intersex/dsd condition	5 (23.8)	4 (26.7)
2. Acknowledging my body resp. the body of my child	3 (14.3)	3 (20.0)
3. Supporting my individual development resp. of my child	5 (23.8)	6 (40.0)
4. Broadening of my thinking	3 (14.3)	2 (13.3)
5. Understanding and emotional support	3 (14.3)	4 (26.7)
6. Community, solidarity and overcoming isolation	3 (14.3)	3 (20.0)
7. Minimising the pressure to decide (e.g., regarding irreversible medical interventions)	3 (14.3)	5 (33.3)
8. I have received important legal information.	3 (14.3)	4 (26.7)
9. I have received important medical information.	5 (23.8)	4 (26.7)
10. I have received important information regarding sex and gender development.	4 (19.0)	2 (13.3)
11. Support in decision-making	3 (14.3)	2 (13.3)
12. Talking with other parents of children with intersex/dsd conditions	1 (4.7)	2 (13.3)
13. Other reasons^d^	3 (14.3)	2 (13.3)^d^

^a^The parents’ responses included 4 × replacing gonads.

^b^As not all parents and all experts of experience have reported previous counselling experiences, the findings refer to those who replied with yes, they have had psychosocial counselling, i.e., 15 of 27 parents replied and 21 of 40 experts of experience.

^c^Example: parent’s answer: “Learning from adults with a dsd who have lived a normal live despite their diagnosis, gives hope that my child will make it too”.

^d^Examples: parents’ answers: “a greater openness towards support groups within the medical system”; “counselling offers for both parents and their child together, not only single consultations with either the parents or the child”.

#### Helpful and missed aspects and contents

Table 2b shows helpful experiences and aspects missed or wished for in the counselling experiences of parents and adults with intersex/dsd conditions. Both groups reported that talking had been helpful, i.e., “talking with other parents” (93%, 14/15), respectively, “talking with other adult experts of experience” (81%, 17/21). Furthermore, receiving “understanding and emotional support” was reported as being helpful similarly in both groups, i.e., by 67% (14/21) of the adult experts of experience and by 60% (9/15) of the parents. Also “broadening of my thinking” and experiencing “community, solidarity and overcoming isolation” were named by far more than half of the participants in both groups. While 60% (9/15) of the responding parents also reported “talking with persons with intersex/dsd conditions” to be helpful, only 38% (8/21) of the adults vice versa found it helpful to talk to parents of children with intersex/dsd.

Among the aspects missed or wished for in the counselling situations they had experienced, respondents of both groups, i.e. 40% (6/15) of the parents and 24% (5/21) of the experts of experience, reported that they had wished for support regarding their individual development resp. their child’s individuality. Minimising the pressure to decide for their child, for instance on irreversible medical interventions, was named by 33% (5/15) of the parents. About a quarter of both groups had missed receiving important medical information and talking with other persons with a intersex/dsd condition. Twenty-seven per cent (4/15) of the parents had wished for receiving understanding and emotional support, as well as important legal information. Twenty per cent (3/15) of them, and 14% (3/21) of the adult patients, had wished for solidarity and overcoming isolation as well as for experiencing acknowledgement of their or their child’s body.

#### Parents’ counselling experiences and preferences, critical situations and topics

Out of the 27 parents who participated in the study, the majority thought medical doctors (81%, 22/27) and peer counsellors (78%, 21/27) were most suited for offering dsd-specific counselling, followed by professional counsellors such as psychologists or social workers (74%, 20/27), midwives (48%, 13/27), psychotherapists (37%, 10/27), sex therapists (19%, 5/27), medical doctors from sex medicine (15%, 4/27) and law experts (7%, 2/27). With regard to the counselling setting*,* “face-to-face” was ranked first when choosing the best way of communication (94%, 16/17), followed by telephone (53%, 9/17), via e-mail (41%, 7/17), web-based care (41%, 7/17) and on meetings (42%, 11/27).

Fiftysix per cent (15/27) of the parents reported to have used psychosocial counselling before. Referring to their experiences in regard to shared decision-making and informed consent processes, 18 parents replied with 50% (9/18) reporting they did “not feel well enough advised before consenting to medical interventions” on behalf of their child.

Only one of the parents, who replied to the item (7%, 1/15), reported that counselling was available when needed. When asked about the specific situations, in which they retrospectively most needed counselling services, parents named the following critical situations: “when I first learned about the intersex condition of my child”, “dealing with everyday situations”, “in childhood, when everything seems to run but questions and insecurities came up; before the beginning of the puberty”. With regard to topics most missed in counselling, “ways of dealing openly with intersex” (40%, 6/15), “sexuality and partnership of my child” (40%, 6/15), “the legal situation” (33%, 5/15) and “limitations and risks of medical interventions” (33%, 5/15) were named most frequently by the parents.

Fifteen parents further replied to the items about the quality of their counselling experiences. Twenty per cent (3/15) reported to have had mostly positive experiences, whereas the majority (80%, 12/15) reported of having had both, positive and negative, counselling experiences.

### Localisation of counselling offers

#### Where to go? Centres and decentralised care

**Fig. 1:**
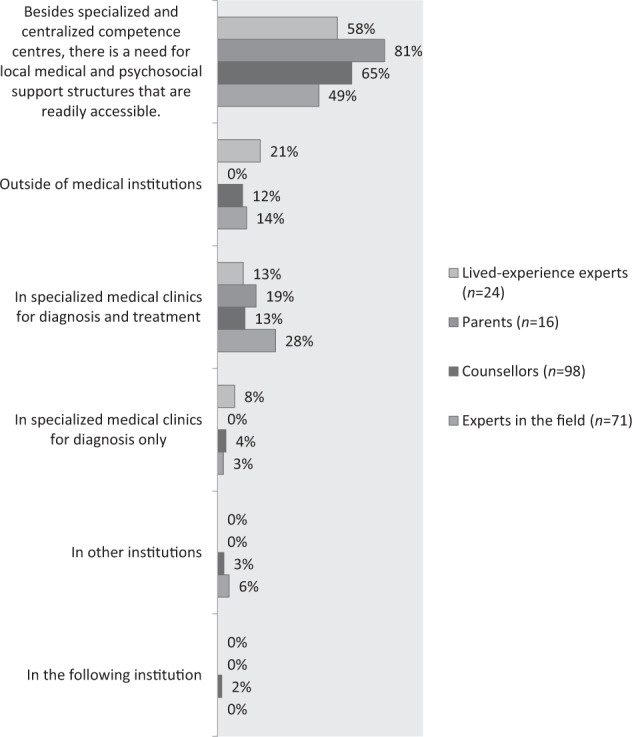
Localisation of counselling offers. Frequency of answers in the subgroups lived-experience experts, parents, counsellors and experts in the field.

In total, 228 participants responded to the question where they find the localisation of multidisciplinary competence centres useful. The results are shown in Fig. 1. Of those, 18% (41/228) thought that specialised medical clinics and hospitals were most appropriate for diagnosis and treatment, whereas 14% (32/228) indicated that these centres should be located outside of medical institutions. Moreover, 4% (8/228) mentioned specialised clinics for diagnosis only (without treatment) and 4% (8/228) preferred other institutions or named, e.g., general counselling centres, centres for sexual counselling, health centres and centres for expertise at University Medical Hospitals. The majority (57%, 131/228) of respondents, however, stressed that besides specialised and central competence centres, there is a need for local medical and psychosocial support structures that are readily accessible. A particularly high proportion within the parent group (81%, 13/16) agreed with this statement.

### Improving care

All participants of the study were asked to (freely) name improvements in counselling structures, services and offers for persons with intersex/dsd that they find necessary. In total, 276 persons (30 lived-experience experts, 16 parents, 173 counsellors and 57 experts) replied to this open, qualitative question that was analysed qualitatively. The contents and identified topics were grouped into the following categories: (1) general needs, (2) increasing quality, (3) increasing quantity and (4) missing knowledge, experience and no assessable improvement necessities. The meaning units of each category were differentiated into further subcategories, which are depicted in Table 3.

**Table 3:** Necessary improvements for counselling and care (qualitative categories).

1. General needs
Education and information, removal of taboos, etc.
2. Increasing quality
2.1 Counselling structures and processes: structuring and networking, low-threshold access, etc.
2.2 Attitude: respectful and sensitive, open, understanding
2.3 Contents: integration into all areas of psychosocial counselling, judicial education, social aspects, etc.
2.4 Qualifications: trainings/specific further education, improvement of vocational training
3. Increasing quantity
Counselling offers, financial resources, etc.
4. No resp. not assessable improvement necessity

These four main categories and subcategories on improvement needs in counselling and support in the field of intersex/dsd are described below. In sum, “counselling structures and processes” within the category “increasing quality” was the subcategory most often mentioned by both respondents in the lived-experience/patients group as well as by parents (33%, 10/30 and 56%, 9/16). The second most frequent topics raised by lived-experience experts belonged to “increasing quantity” (23%, 7/30). In contrast, the parents chose topics related to the category “qualification” second most often (44%, 7/16). Within the counsellor group, the highest proportion of the chosen topics belonged to the category “general needs/necessities” (35%, 61/173). One-third of their topics belonged to the subcategories “counselling structures and processes” (33 %, 57 of 173) and “qualifications” (30%, 52/173), respectively, and hence played a crucial role. The responses of the experts in the field were predominated by the category “counselling structures and processes”.

### General needs

The overall picture of answers was predominated by the topic of education/information. Some examples are: “more public relations” (one counsellor) or “first and foremost education of the population” (one expert). Furthermore, the following topics appeared as general necessities: (1) removal of taboos—“the subject must lose its taboo stigma in order to ensure an open way to deal with it” (one counsellor); (2) sensitisation—“higher sensitivity of all occupational and population groups” (one counsellor); (3) freedom of choice of one’s sex—“freedom of choice of one’s sex far beyond puberty! General toilets in all public areas, because I am a man and a woman” (one lived-experience expert).

### Increasing quality

Within the category of “increasing quality”, the following subcategories appeared: (1) counselling structures and processes, (2) attitudes, (3) contents and (4) qualification.

#### Counselling structures and processes

The subcategory “counselling structures and processes” was dominated by answers that focused on structuring and connecting counselling offers as well as easily accessible counselling offers. Among others, the following respondents pleaded for such improvements: “Move away from counselling jungle” (one lived-experience expert); “exchange and connecting” (one counsellor); “networks/contact persons/umbrella organisations that are more present” (one expert); “Initial clear contact persons and structuring of counselling and supply offers” (one expert); “low-threshold access to knowledge of counselling offers within the population” (one counsellor); “more nationwide supply, especially in rural areas” (one counsellor).

Moreover, specialisation and independence were further recurring subcategories of the “counselling structures and processes”. One lived-experience expert, e.g., mentioned the necessity of “more specialised counselling centres” and an expert identified “more specialised colleagues” as necessary. With regard to the issue of independence, one parent wrote: “There should be at least one counselling centre per Federal State that works independently from doctors”. Another parent wrote: “There should be more counselling centres within Germany with qualified counsellors that work independently from medicine, but of course in cooperation with medical counsellors”.

Several times, the demand for (1) interdisciplinary work (“interdisciplinary team that also works this way” (one lived-experience expert)); (2) peer-to-peer counselling by inter*-persons (“I only perceive intersex persons as suitable for counselling intersex persons” (one lived-experience expert), inter*-specific counselling offers through peers (one expert)) and (3) online offers (“particular online counselling services with a central counselling office” (one counsellor)). Some persons mentioned aspects such as continuity, medical competence centres as well as requirement analyses. For instance, one counsellor wrote: “probably continuous support, if desired”. Another counsellor said: “requirement analyses (How do affected persons perceive it?)”. One lived-experience expert wrote: “There should be medical competence centres, the people who work there should know that there is more than man and woman, and they should counsel and treat accordingly”.

#### Attitudes

Within the subcategory “attitudes”, improvement suggestions and values such as respect, sensitivity, understanding and openness came up, as illustrated by the following answers: “widening the supply for a non-pathologizing view” (one counsellor); “dealing with this matter needs to become more openly, especially in maternity hospitals, at paediatricians and registry offices” (one counsellor); “physicians should not treat us as an attraction, but as normal” (one lived-experience expert); “Better understanding of the shocking and helpless situation of PARENTS after hearing about the dsd diagnosis which should not be tried to be solved by treating the CHILD. Being able to stand grief, rethinking and waiting” (one parent).

#### Contents

Among others, incorporating the topic of intersex in counselling offers with other focuses was demanded as content-related improvement need. For instance, this topic should be also considered within the context of pregnancy counselling, youth welfare and family and school counselling. Other respondents increasingly perceived the consideration of advantages and disadvantages of medical treatment, education about juridical aspects, and more consideration of social aspects as well as a reduction of technical terms as necessary.

#### Qualification

Among all replies, the demand for “qualification” was strongly expressed. Training and specific (further) educations were recurrently described as great needs and necessities that are currently lacking. Among qualifications in terms of “hard skills” in the form of expert knowledge, especially one parent brought up the topic of “soft skills”: “There is an urgent need for trainings how to inform and educate affected persons about their diagnosis, respectively. If this very first moment goes wrong, processing the diagnosis develops negatively.” Occasionally, not only possibilities to receive additional qualifications were mentioned, but also an improvement of the (basic) education (“People should be better educated” (one lived-experience expert), “embedding the subject within medical studies” (one counsellor)).

### Increasing quantity

Finally, a high proportion of the respondents’ answers included aspects on “increasing quantity”. The necessity to generally augment counselling offers can be, e.g., expressed by the following answer of the parent group: “There should be counselling services in the first place, in South Germany there is a counselling desert.” The need to increase financial resources (e.g., secure financing for already existing offers, funding of particular offers and covering of travel costs), an increase of counselling centres, augmentation of qualified personnel and/or extension of temporal capacities were mentioned repetitively. “Interest for the subject” (counsellor) was another reply that belonged to the category “increasing quality”.

### Missing knowledge or experience, absent improvement needs

The fourth residual category subsumes those answers implying “no or not assessable improvement necessities”, including missing knowledge or experience. For instance, one counsellor wrote: “I do not know”. Other persons mentioned that they never met a person with the according counselling need.

## Discussion

The aim of this study was to explore experiences and improvement needs in counselling and support structures in the field of intersex/dsd on different levels and as evaluated by different groups of experts, such as parents of children with intersex/dsd conditions, adult experts of experience themselves, as well as counsellors and experts in the field. The results show a great demand for more contact points and counselling centres for intersex/dsd and thereby support earlier and recent calls for improvement [12, 16]. Especially the lack of counselling experiences with individuals with intersex/dsd or their parents of the largest group of participants, the professional counsellors, shows that there is still a barrier and little knowledge on intersex/dsd outside of specialised centres or reference networks. This central finding hints at the necessity to speak more openly and informative about intersex/dsd and to communicate the need for psychosocial caregivers who are willing to become more informed and educated in the field. While psychosocial counsellors outside the medical system have started to provide counselling and support to other minorities in the field of sexual and gender medicine, e.g. for transgender persons and or patients with gender dysphoria, the field of intersex/dsd is yet to be discovered and appreciated by these professionals, particularly as incidences of intersex/dsd conditions (as an umbrella term) are higher than those of gender dysphoria and gender incongruence [35].

The basic result on counselling experiences further shows a gap between receiving psychosocial and medical care in the group of adult lived-experience experts, who had received less psychosocial care than medical interventions. The findings on helpful counselling experiences and aspects missed by those participants who reported to be psychosocial service users having had counselling experiences, highlight important topics that need to be addressed in a psychosocial counselling process in the field of intersex/dsd. Psychosocial care in form of a continuous process in all phases of dsd healthcare is described as a mandatory element to be provided by multidisciplinary teams (MDT) inside and outside of reference networks and centres according to the German guidelines that were published in 2016, a year after the conduction of this study [21]. The central needs and aspects in counselling as shown in the study presented included talking with other experts of experience or parents who went through similar experiences, but also receiving emotional support and solidarity. Tasks of psychosocial care that can be inferred are to provide understanding, an appreciation for the individual body and development, helping to overcome isolation, as well as providing relevant medical information.

Regarding the localisation of specific intersex/dsd counselling, more than half of the respondents in the parents and adult experts of experience group reported travelling far distances to reach counselling services. Besides multidisciplinary competence centres, the majority of participants thought that regional medical and psychosocial support structures are important as well. Within the parents’ group, this demand was especially high. The results suggest that within the parent group, there is a high need for proximity and accessible support structures. Parents who had taken advantage of intersex/dsd counselling services further regarded an open communication and broadening their own thinking as important. Talking with other parents and receiving emotional support have been helpful aspects. The support wished for included minimising the pressure to decide on behalf of their child on irreversible medical interventions.

The results of the qualitative analysis regarding the perceived needs for improvement regarding present care structures emphasised a high need for a better interplay between already existing structures and offers. Not only a lack of counselling and advice services, but also the deficient cooperation between counselling structures and their stakeholders and relevant persons involved, as well as the lack of knowledge and familiarity with the topic became apparent. Such gaps and deficits may further result in an insufficient referral of help-seeking persons to the appropriate services. Education of the general population appeared as an important topic too, which holds true especially within the counsellor group but also for the general public, as the birth of an intersexed child can occur in every family, but hardly any future parents are well informed about the fact that they might not only become parents to a girl or a boy but also to an inter-child.

Our findings imply that improving the quality and the quantity of psychosocial care in the field of intersex/dsd remains a central goal within and outside the healthcare system. They also allow to specify the bifocal task of psychosocial care, counselling and support, which equally includes providing rational and understandable information on the one hand as well as providing emotional holding that enables processing information, symbolising the new information and developing an internal representation of intersex/dsd and what that may mean for everyday life.

Nevertheless, clinical experience shows that sometimes caregivers experience a reluctance or fear of patients towards making use of psychosocial care and support offers, particularly of adolescents or parents who have difficulties accepting an intersex/dsd diagnosis. This might be an expression of the problem that psychosocial care is not yet a self-evident, “normal”, element of health just as a matter of course like somatic interventions; further it also might be an expression of the lack of providing a secure, accepting and acceptable language and terminology for both, intersex/dsd conditions and people with intersex/dsd, as well as the psychosocial staff, which might be able to help with acceptance and shame management for those involved [12].

A more severe obstacle is the lack of well-educated staff and personnel, as shown by the study of Kyriakou et al. [24]. Their study shows that 60% of the MDT participating in their research do lack psychosocial caretakers and other relevant professionals in their teams. Without these staff members who are able to meet the needs of bifocal counselling, providing and repeating information and emotional support at the same time is necessary to support parents, families and patients. Allowing and supporting individual development was raised as a central task of intersex/dsd counselling. From a developmental psychology perspective, allowing and promoting individual psychosexual development as an individual with an intersex/dsd variation is the prerequisite for developing trust in oneself and one’s environment and thus for becoming a self-confident and responsible individual, not despite but with an intersex/dsd condition.

Learning from other empowerment and peer-to-peer concepts, such as initially developed by psychiatric patient groups, the exchange between lived-experience experts, relatives and experts should be promoted to enhance mutual understanding of the different perspectives. Peer-to-peer experts could get in touch with students to open up for exchange and hence promote anti-stigmatisation as well as counteract “exotisation”. In this way, there should be the possibility for a constructive encounter between experiential and professional knowledge [36]. However, the integration of different stakeholders, from activism, support and self-help into professional structures should ensure that its principles of non-commerciality, freedom, voluntariness and equality are maintained. Moreover, promoting self-help and mutual support and empowerment should not be instrumentalised or misused as easy and cheap solution to supply bottlenecks [37]. Rather, peer experts should be funded as well as professional experts, either in care or in research as it has been called for from different sides [38, 39]. Here, as in the general context of intersex, cautious and sensitive working and negotiation processes are necessary. If this succeeds, there will be a closer cooperation with the self-help and support groups which means a great benefit for all “affected persons” [36].

### Limitations

The short data collection period limited the recruitment methods. However, a high number of participants took part in the study. The proportion of lived-experience experts and parents was not as large as desired. The external validity of the study cannot be guaranteed which is, however, almost impossible with rather rare or unknown phenomena. Unfortunately, only two midwives participated, which means that an important group is underrepresented within the sample. Moreover, peer counsellors were not considered as target group although this group could have proven valuable insights. Hence, a follow-up study and repetition of the survey should investigate and integrate a higher proportion of midwives and peer counsellors. The repetition would also meet another limitation, which is the age of the data, that was collected already in 2015. As currently a paradigm change in intersex/dsd care is being observed and reported, an up-to-date data collection should be able to document any change in the status quo and to check whether new models of care that are aiming at overcoming the deficits and discursive gaps between the disciplines involved have yielded or contributed to an improvement of sustainable care [20–22, 27, 28].

Furthermore, to use stronger qualitative methods in future research would allow to investigate the present results regarding needs and deficits in more detail. Moreover, a follow-up study with a longer time frame is desirable, not only in order to gain an even more holistic insight, but also to study whether change has happened and structures have been implemented or not.

## Conclusions

The results of the present study reflect the situation five years ago. Nevertheless, they outline necessary improvements, experiences and needs. They point out tasks for psychosocial care and deficits in current counselling structures and support services for persons with intersex/dsd and their parents. Next to specialised counselling offers, local provision of comprehensive support is perceived as a crucial improvement and necessary basis. Moreover, supporting self-help structures and improving networking and cooperation between already existing counselling services are highly needed. A useful implementation could be the sensible interplay of digital and face-to-face, professional and peer counselling, allowing for mutual learning, support and empowerment.

The findings indicate that parents of children with intersex/dsd conditions have a high need for accessible counselling structures but that there is a lack of specific support options. Improvement should start with training more professionals in offering counselling for families with intersex/dsd conditions. In addition, although the findings reflect parents’ experiences from the year 2015 and before, they allow to imply a shift in practice towards an increase of psychosocial care. While nearly 40% of the parents reported that their children had received medical interventions, the same number reported to have received psychosocial counselling. In contrast, in the group of adults who reflected on the medical treatment they had received in response to their intersex/dsd, 30% appear not to have received any psychosocial care.

Though the results reflect a past situation, both improvements and needs of improvements become visible. While a shift in thinking and practice has been described, further studies are now needed in order to understand the specific effects of those changes in care in the meanwhile [26], but also to focus on different target groups and their counselling experiences, like parents, children, adolescents and adults over life span [40]. A repetition of the survey is encouraged to identify change and to check how the narrative of the paradigm change is transformed into practice and how it is perceived by service users and providers at the same time. In addition, further studies are needed to follow-up possible changes in practice in different settings, not only reference centres but in all medical and other institutions responsible for dsd care, to get a real picture.
